# Towards a Mobile Gait Analysis for Patients with a Spinal Cord Injury: A Robust Algorithm Validated for Slow Walking Speeds

**DOI:** 10.3390/s21217381

**Published:** 2021-11-06

**Authors:** Charlotte Werner, Chris Awai Easthope, Armin Curt, László Demkó

**Affiliations:** 1 Spinal Cord Injury Research Center, University Hospital Balgrist, 8008 Zurich, Switzerland; Armin.Curt@balgrist.ch (A.C.); laszlo.demko@hest.ethz.ch (L.D.); 2 Rehabilitation Engineering Laboratory, Department of Health Sciences and Technology, ETH Zurich, 8008 Zurich, Switzerland; 3 Cereneo Foundation, Center for Interdisciplinary Research (CEFIR), 6354 Vitznau, Switzerland; chris.awai@cereneo.foundation

**Keywords:** gait analysis, inertial sensors, IMU, spinal cord injury, rehabilitation, clinical assessment, wearables, e-health

## Abstract

Spinal cord injury (SCI) patients suffer from diverse gait deficits depending on the severity of their injury. Gait assessments can objectively track the progress during rehabilitation and support clinical decision making, but a comprehensive gait analysis requires far more complex setups and time-consuming protocols that are not feasible in the daily clinical routine. As using inertial sensors for mobile gait analysis has started to gain ground, this work aimed to develop a sensor-based gait analysis for the specific population of SCI patients that measures the spatio-temporal parameters of typical gait laboratories for day-to-day clinical applications. The proposed algorithm uses shank-mounted inertial sensors and personalized thresholds to detect steps and gait events according to the individual gait profiles. The method was validated in nine SCI patients and 17 healthy controls walking on an instrumented treadmill while wearing reflective markers for motion capture used as a gold standard. The sensor-based algorithm (i) performed similarly well for the two cohorts and (ii) is robust enough to cover the diverse gait deficits of SCI patients, from slow (0.3 m/s) to preferred walking speeds.

## 1. Introduction

Spinal cord injury (SCI), either caused by a trauma (e.g., accident) or disease (e.g., tumors), leads to permanent changes in the central nervous system. Depending on the severity and location of the injury, the symptoms vary widely: the patients might have a partial or complete loss of sensory function and motor control in the legs, arms, or whole body [1]. In particular, an incomplete SCI refers to remaining sensorimotor functions below the injury level, which can allow some patients to regain walking function despite their injury [2]. As independence in mobility is a crucial factor for performing daily life activities and for participating in society, regaining walking function is a common goal in most rehabilitation programs for these patients [3].

An accurate gait assessment during rehabilitation can give an insight into the recovery of motor functions and help with clinical decision making due to impairment specific training [4]. However, in a typical clinical routine, only simple gait tests such as the six-minute walking test are performed [5]. The outcome of these tests is the distance covered or the time to perform the test. Therefore, only the average walking speed of the patient is assessed. On the other hand, in research, a detailed gait analysis is conducted in gait laboratories, which are usually equipped with complex setups such as force plates and motion capture systems [6]. Here, the outcome is a comprehensive analysis of the gait kinematics and joint kinetics. However, these gait laboratories are costly, postprocessing of the data is time-consuming, and the measurements are restricted in both space and duration.

Wearable inertial sensors could potentially be a balance between simple but less detailed clinical assessments and costly but highly informative gait analyses in laboratories. An advantage of the inertial measurement units compared to gait laboratories is that the sensors are small, affordable, and can be attached to different body parts according to the use case [7]. A potential application of these inertial sensors is to complement the aforementioned clinical gait tests by providing, in addition to the walking speed, information on the gait pattern and thus gait deficits of the patient. The benefit of using inertial sensors during the six-minute walking test has already been demonstrated for neurological diseases including multiple sclerosis [8,9,10,11], Parkinson’s disease [12] and stroke [13]. Furthermore, wearable sensors carry the promise for long-term monitoring of gait speed and quality outside of laboratory settings, opening the potential to target remote interventions for individual patients.

In the past decade, a considerable amount of algorithms arose to investigate gait deficits with inertial sensors, focusing on the gait of elderly, stroke, multiple sclerosis, or Parkinson’s disease [14,15,16,17]. The typical approach of such algorithms is to detect gait events like initial and final foot contact, then estimating the sensor trajectory for each stride. Based on this, spatio-temporal parameters like step duration, gait phases, and walking speed can be calculated. Various methods exist to extract these parameters, which differ in terms of complexity, robustness, and computational effort [18,19].

To the best of our knowledge, up to now, no validated algorithm exists to reliably extract these spatio-temporal gait parameters of SCI walking from shank-mounted inertial sensors. Jasiewicz et al. [20] presented a method to extract only the initial and final foot contact, and Tong et al. [21] validated their algorithm solely for one single patient, which is not representative of the wide variety of gait patterns found in this patient population. A possible reason for the lack of algorithms for SCI patients could be that the prevalence of SCI is lower compared to, e.g., stroke. Furthermore, people with SCI do not have a pathognomonic walking pattern like, for example, patients with Parkinson’s disease. On the contrary, the gait deficits of SCI patients vary widely depending on the degree and level of the injury [22] often inducing compensatory movement patterns. Thus, algorithms that rely on fixed thresholds are prone to fail for these pathological gait patterns.

Furthermore, algorithms are often validated only for normal walking speeds of around 1 m/s. However, severely affected patients that are only able to walk indoors under supervision usually have a much slower average walking speed of ~0.34 m/s [23]. Therefore, it is necessary to validate such algorithms for slow walking speeds so that they can be used reliably in a clinical setting for patients with low functional ambulation.

We here propose a sensor-based gait analysis for SCI that derives typical spatio-temporal gait parameters from inertial sensors attached to both ankles. Our goal was to focus on a minimal but still clinically accurate setup that is non-obtrusive for daily life applications, easy to handle, and can have the potential to be integrated into the daily clinical routine. In contrast to typical sensor-based gait analysis found in literature, where the detection of steps relies on fixed thresholds, we here propose a method that adapts these thresholds based on individual gait profiles. We hypothesize, that our algorithm is more robust against variable gait patterns and variable walking speeds. By validating the sensor-derived gait parameters with a gold standard, we demonstrate that the proposed algorithm is robust enough to be applied for the diverse gait deficits of SCI patients, from slow to preferred walking speeds.

## 2. Materials and Methods

### 2.1. Subjects

This study’s participants (>18 years old) were either patients with a chronic SCI or neurologically unimpaired subjects. The patients were recruited from the patient database of the University Hospital Balgrist. Patients with all neurological levels of injury were included if they (i) were able to stand without physical assistance for more than 120 s, and (ii) had preserved segmental and cutaneo-muscular reflexes in the lower limbs. Patients with current orthopedic problems, psychological disorders, or neurological impairments other than SCI were excluded from the study. Participants for the control cohort could be included if they did not have any orthopedic problems affecting gait. In total, ten SCI patients and 17 healthy controls were recruited for this study.

Each participant was informed about the study procedure and risks before they participated in this study. The study was conducted in accordance with the Declaration of Helsinki, and the protocol was approved by the ethical committee of the canton Zurich (KEK-ZH No. 2017-01780) and by the Research ethics committee of ETH Zurich (EK No. 2018-N-80).

### 2.2. Study Protocol and Data Collection

For the SCI subjects, a clinician assessed the American Spinal Injury Association Impairment score (AIS score) [24]. This AIS score includes assessing motor function in terms of the upper (UEMS) and lower (LEMS) extremity motor score and sensory function in terms of pin prick and light touch sensation. A combination of the sensory and motor function determines the neurological level of injury (NLI). In addition, the Spinal Cord Independence Measure (SCIM) was assessed [25]. For this study, we focused on the mobility domain of the SCIM, including ‘room and toilet mobility’ and ‘indoors and outdoors mobility’, resulting in a maximum achievable score of 40. Demographic information like age, height, body mass index (BMI), and sex were collected for all participants. Furthermore, the walking pattern of the patient was qualitatively described by a physiotherapist.

**Figure 1:**
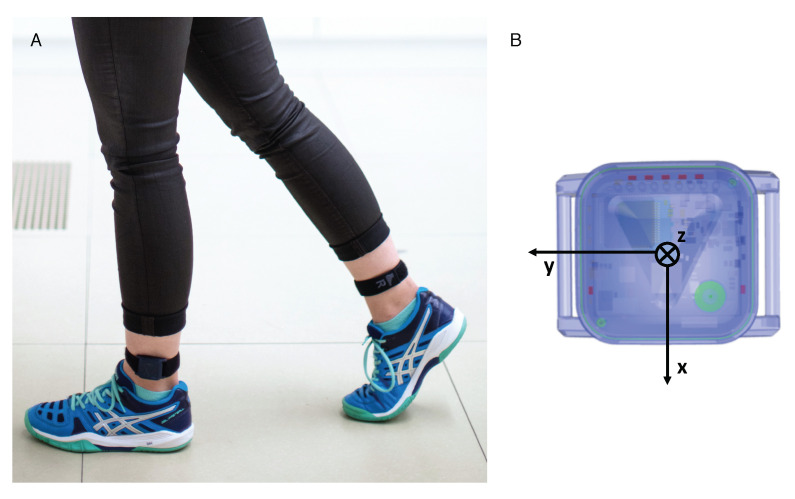
Subject wearing sensor modules attached laterally above each ankle (**A**). Schematics of the sensor module with its coordinate system (**B**).

Two inertial sensors were attached to the participants’ ankles above the lateral malleolus with flexible straps as shown in Figure 1A. The sensor modules used for the study have been developed as part of the ZurichMOVE project [26]. The main components of the modules (35 × 35 ×12 mm, 18 g) include a tri-axial accelerometer, a tri-axial gyroscope, and a tri-axial magnetometer recording with a sampling rate of 200 Hz. As the magnetic field is often distorted indoors, the magnetometer data was omitted from the data analysis. The local coordinate system of the sensor module is depicted in Figure 1B.

The measurements were performed in the Gait Real-time Analysis Interactive Lab (GRAIL, Motekforce Link, The Netherlands), widely accepted as a gold standard for gait analysis. The GRAIL is a compact experimental lab for gait analysis with a treadmill, a motion capture system, and three stationary cameras. The instrumented dual-belt treadmill (V-Gait Dual Belt, Motekforce Link, The Netherlands) has two integrated force plates, which measure the ground reaction forces in three dimensions with a sampling frequency of 1000 Hz. One reflective marker was placed on each sensor and was tracked by ten cameras (Vero, Vicon Motion Systems, United Kingdom) at a sampling frequency of 100 Hz.

Synchronization between the GRAIL and inertial sensors was achieved by placing an additional sensor module on top of a piezo element, which was connected to the trigger line of the motion capture system. The trigger induced vibration of the piezoelectric material during the measurements, which was captured by the additional sensor module. Time synchronization between the three sensor modules was achieved by a master-slave configuration using Bluetooth Low Energy.

Before the first measurement, each participant became familiar with the test environment and the procedure by walking on the treadmill for about 5 min as recommended by Meyer et al. [27]. During this familiarization phase, the speed levels increased starting from 0.3 m/s in steps of 0.1 m/s to determine the preferred walking speed of the patient. The patient had to give feedback as soon as the speed level exceeded his preferred walking speed. For SCI patients, the data of three different speed levels have been collected: preferred walking speed, 0.5 m/s, and 0.3 m/s. The measurement of each speed level lasted for 180s. These levels cover a broad range of walking speeds, including slow walking. More specifically, the speed ranges were selected to represent sub-community and community ambulators, and to ensure that all participants would be able to complete the task. Healthy participants were only measured at the speed of 0.5 m/s for 180 s to have a comparable condition between the two cohorts of the study. All participants wore their normal street shoes for the experiment.

### 2.3. Gait Analysis

#### 2.3.1. Definition of Gait Parameters

**Figure 2:**
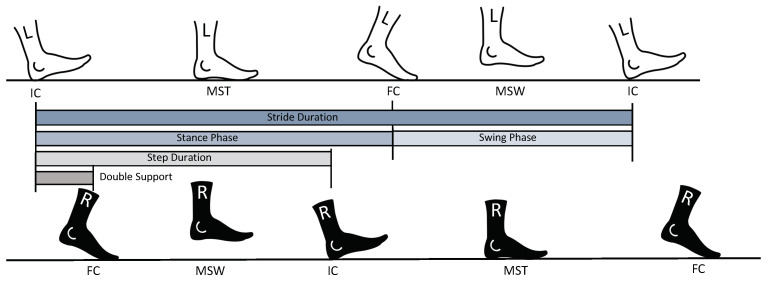
Schematic of the gait events initial contact (IC), final contact (FC), mid-swing (MSW), and mid-stance (MST) of a complete gait cycle and the corresponding gait phases.

Walking is typically segmented into individual gait cycles and further described by temporal gait parameters, which are depicted in Figure 2. A gait cycle corresponds to one stride, which starts with the initial contact (IC) of one foot and ends when the same foot contacts the ground again (following IC). The stride can be divided into a stance phase, where the foot is in contact with the ground, and a swing phase, where the foot is not in contact with the ground. The transition from stance to swing phase is initiated with toe-off. As some patients do not have a typical toe-off, we refer to this gait event by the final foot contact (FC). The double support phase corresponds to the period when both feet are on the ground, namely, from the IC of one side to the FC of the other side. The step duration starts with the IC of one side and ends with the IC of the other side. Other gait events relevant for the analysis are mid-swing (MSW) and mid-stance (MST), defined as the midpoint of the swing phase and stance phase, respectively.

**Figure 3:**
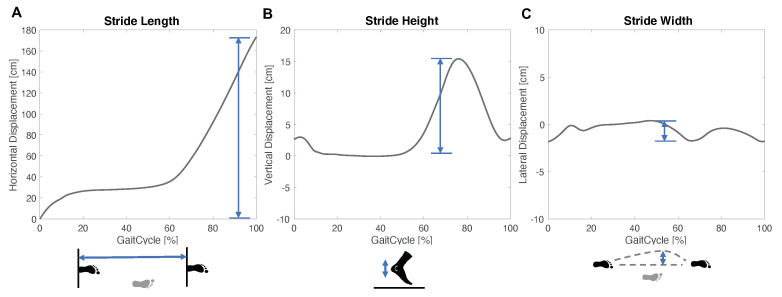
Schematic of the spatial parameter for one gait cycle, which is defined from initial contact to initial contact of the same side.

Spatial parameters describe the displacement of the foot during a gait cycle and are depicted in Figure 3. The stride length is defined as the maximum displacement of one foot in the movement direction within a stride. The stride width and stride height correspond to the lateral and vertical displacement range during a gait cycle, respectively.

#### 2.3.2. Sensor-Derived Gait Parameters

The following data processing workflow has been developed to extract typical spatio-temporal gait parameters from the 3D accelerometer and gyroscope data of the shank-mounted inertial sensors.

First, the data are segmented into individual gait cycles. For this purpose, a fast Fourier transform (FFT) is applied to the angular velocity perpendicular to the sagittal plane ($\omega_{z}$) to obtain the frequency spectrum. The first main frequency component ($f_{max}$) corresponds to the periodicity of the cyclic movement of walking. Thus, the inverse of $f_{max}$ corresponds to the average gait cycle duration ($T_{cycle}$):

(1) $$T_{cycle}=\frac{1}{f_{max}}\;with\;f_{max}=max\left(FFT\left(\omega_{z}\right)\right)$$

Individual gait cycles are identified as prominent local peaks of $\omega_{z}$, corresponding to mid-swing (MSW). Instead of using fixed thresholds for the minimum peak height and the minimum distance between two consecutive peaks, these thresholds are adapted for each measurement for algorithm robustness across different walking speeds and cadences. The minimum peak height is defined as 20% of the 99*^th^* percentile of $\omega_{z}$ and the minimum peak distance as 50% of $T_{cycle}$.

Accurate identification of initial foot contact (IC) and final foot contact (FC) is paramount for further calculating gait parameters, as these serve as anchors for the temporal parameter definition. These gait events are detected by local peaks of the acceleration data in the anterior–posterior direction (a${}_{y}$) and $\omega_{z}$. Similarly, as before, the window in which to search for these peaks is adapted based on $T_{cycle}$. Because the IC typically occurs at 20% of the gait cycle after MSW, IC is defined as the local maximum in a${}_{y}$ within 5% to 45% of $T_{cycle}$ after MSW. The FC is defined as the midpoint between the minimum in $\omega_{z}$ and the maximum in a${}_{y}$ within −35% to −5% of $T_{cycle}$ before MSW. Finally, mid-stance (MST) is identified as the maximum of $\omega_{z}$ within the IC and the following FC. The temporal gait parameters are then computed as defined in Section 2.3.1.

The spatial parameters were computed from the 3D trajectory of the sensor during each stride. To obtain this trajectory, the orientation of the sensor in space was estimated by the magnetometer-free orientation estimation algorithm developed by Seel et al. [28]. Using this estimated orientation, the acceleration data were transformed from the local coordinate system of the sensor, which rotates with the movement of the sensor, into a fixed coordinate system. This is necessary because the acceleration data consists of both movement and gravitational acceleration. In the fixed coordinate system, the gravitational component could be easily removed from the vertical axis, which corresponds to the direction of earth’s gravity. After receiving the pure movement acceleration, the acceleration data is integrated for each stride from MST to MST with a trapezoidal integration. As the sensor data is contaminated with noise, an integration of this thermo-mechanical noise results in a first order drift for the velocity and a second order drift for the displacement. Therefore, the following boundary conditions were introduced to correct for this drift: Instead of the often used zero-velocity update, the initial and final velocity values of each stride are estimated from the angular velocity at MST multiplied by the distance between the sensor and the ankle joint as proposed by Li et al. [29]. This distance is estimated to be 10 cm. In addition, for abnormally long strides (>2.5 s), a first-order high-pass Butterworth filter with a cut-off frequency of 0.0002 Hz has been introduced to reduce the effect of drift. Moreover, without using the magnetometer data, the heading direction is undetermined and would result in a drift around the vertical axis. Therefore, the heading angle was chosen to be fixed to the main movement direction, which is estimated for each stride according to Trojaniello et al. [30,31]. In brief, the main movement direction was determined by the direction of the velocity during the swing phase within the horizontal plane. Then, the 3D velocity data for each stride is integrated a second time to obtain the 3D sensor trajectory for each stride. Finally, the stride length, width, and height are calculated as defined in Section 2.3.1.

#### 2.3.3. Gait Parameters from Gait Laboratory

The true initial and final foot contact events have been derived from the data measured by the force plates of the split-belt treadmill. The ground reaction forces of the vertical direction were filtered with a low-pass fir filter with a cut-off frequency of 30 Hz. Sequences of a vertical ground reaction force smaller than 30 N and shorter than 0.2 s were equally set to zero. Every sequence with non-zero values was treated as a stance phase. The beginning and end of each stance phase were identified as the IC and FC, respectively. For each stride, its duration, the swing phase, the double support phase, and the step duration have been computed as defined previously. Whenever a participant stepped in the middle of the dual-belt treadmill, a signal was generated on both the left and right force plate, leading to an indistinguishable swing phase from the stance phase. As this contaminated the ground truth measurement, these strides had to be excluded. Trials with fewer than ten valid strides in total were excluded from the analysis. The spatial gait parameters were derived from the data of the reflective markers attached to the sensors. The 3D displacement of the markers was segmented into stride sequences from the ground-truth IC to the following IC of the same side. The stride length was calculated as the difference between the anterior–posterior positions of the marker in the direction of the treadmill at the beginning and end of each cycle, plus the distance the treadmill belt moved during that time span. The stride height was computed as the maximum displacement in the vertical direction during the swing phase compared to MST. The stride width was defined as the maximum lateral derivation of the foot from the straight line.

### 2.4. Statistical Analysis

To characterize the walking pattern of the SCI patients and healthy controls, the means and standard deviations of the spatio-temporal gait parameters were computed for the different speed conditions. In addition, the intra-subject variability was obtained as the average of the within-subject standard deviations.

The differences between the sensor-derived gait parameters and the ground truth values from the gait laboratory were computed for each gait cycle to investigate the performance of the algorithm. This difference is referred to as the error in the following. For each participant the average error over all gait cycles was computed for the left and right side. As some of the patients had an asymmetric gait pattern, all participants’ left and right sides were treated separately to not average out the effect of the more affected side. Furthermore, the mean relative error was obtained as the mean error divided by the corresponding ground truth gait parameter. For the initial and final contact detection no mean relative error can be computed, because the error was derived as the difference in timing and not as the difference to an actual gait parameter. In addition, no mean relative error was reported for the stride width and stride height, because the ground truth values were in comparison to the errors rather small leading to inflated values when the error was divided by values close to zero.

As summary metrics, mean and standard deviation values were reported for the different speed conditions and cohorts. A Bland–Altman analysis was performed on the trials of all speed conditions and cohorts to investigate whether the algorithm is affected by any measurement bias [32]. For this, the mean error and the 95% limits of agreement between the sensor-derived gait parameters and the ground-truth values from the gait laboratory were computed. Due to the non-normality of the data, an unpaired two-sample Wilcoxon test was applied to reveal the difference between the algorithm’s performance on the two different cohorts of this study. Furthermore, a Friedman rank-sum test was performed to investigate the effect of speed on the performance of the algorithm. The Wilcoxon test and the Friedman rank-sum test used the median and the interquartile ranges of the errors and a significance level of 0.05.

## 3. Results

### 3.1. Subjects

The data of 26 participants of this study (50% female) were included in the analysis. For one patient, there was no trial with more than ten valid strides. Therefore, this patient was excluded from the further analysis. For all the remaining nine SCI patients and 17 healthy controls all trials had a sufficient number of strides and thus were all included. The average age of the control group was younger (27.6 ± 2.9 years) than the age of the patient group (59.6 ± 7.4 years). However, the SCI patients and the healthy participants had a similar height of 172.8 ± 7.5 cm and 170.6 ± 9.5 cm, respectively, and similar BMI of 22.7 ± 4.4 kg/m${}^{2}$ and 22.0 ± 2.8 kg/m${}^{2}$, respectively. The lesion completeness on the AIS score was ranked as D (sensory and motor incomplete) for all SCI patients, with a NLI range of C7 to L3. More specifically, the patients had an average lower extremity motor score (LEMS) of 47.6 ± 4.9 out of 50, indicating moderate motor deficits. In terms of sensory deficits, the patients achieved an average pin prick score of 40.6 ± 14.6 and an average light touch score of 45.4 ± 9.5 out of a maximum score of 56. All patients suffered from sensory or motor deficits of different degrees, except for patient SCI06. Even though this patient achieved a full score in the LEMS, light touch, and pin prick assessment, he reported difficulties in postural control and thus balancing during walking. An average score of 37.2 ± 4.2 out of 40 was assessed for the mobility domain of the Spinal Cord Independence Measure (SCIM) score. Details of the demographics and clinical scores of the SCI patients are reported in Table 1.

### 3.2. Walking Characteristics

The walking pattern of healthy controls and SCI patients was characterized using the ground-truth spatio-temporal parameters derived from the gait laboratory. In total, 5590 gait cycles of the SCI patients and 2924 gait cycles of the healthy controls were included in the analysis, and the results are reported in Table 2. Strides had to be excluded when the participants stepped in the middle of the dual-belt treadmill.

As the majority of these spatio-temporal parameters are speed-dependent, only the gait parameters of the 0.5 m/s speed condition were compared between the two cohorts. Both participant groups walked with a similar stride length, resulting in a similar stride duration driven by the fixed speed of the treadmill. Similarly, comparable results have been obtained for the remaining gait parameters. However, the standard deviation of all gait parameters of the SCI patients exceeded the standard deviation of the control group indicating a higher inter-subject variability. Similarly, a higher intra-subject variability was obtained for all gait parameters in the SCI cohort compared to healthy controls. For SCI patients, this intra-subject variability was lower than the inter-subject variability for all gait parameters, except for the stride width.

The SCI patients had a preferred walking speed of 0.76 ± 0.17 m/s. By comparing the gait parameters of the 0.5 m/s and 0.3 m/s speed condition to the preferred speed, a longer stride duration, shorter stride lengths, and decreased stride heights were obtained. In addition, the percentage of the swing phase decreased with a decreased walking speed, whereas the relative stance phase and thereby the double support phase increased. Furthermore, the intra-subject variability and the inter-subject variability were with few exceptions lower or equal in the preferred walking speed condition compared to the 0.3 m/s and 0.5 m/s walking speed for all gait parameters.

The patients showed a range of different walking deficits, which were qualitatively described by a physiotherapist. Patients SCI01, SCI02, SCI04, and SCI05 were walking with a drop foot on either one or both sides. An ataxic walking pattern was observed for patients SCI01, SCI03, and SCI09. Patients SCI01, SCI03, SCI06, and SCI09 suffered from spasticity or an increased muscle tone during walking. Moreover, patients SCI03 and SCI08 had a decreased stability and balancing issues. Patient SCI05 had an asymmetric walking pattern due to a left sided tetraparesis. This induced a circumduction of the left leg and toe walking of the left side as a compensatory strategy. Only patient SCI07 showed a normal walking pattern with no obvious walking deficits.

### 3.3. Validation of Sensor-Derived Gait Parameters

The error of the typical spatio-temporal parameters was calculated between the GRAIL-estimated ground truth and the results of the proposed algorithm. Neither missed nor extra gait cycles generated by the proposed algorithm were observed.

#### 3.3.1. Comparison of Errors between Cohorts at 0.5 m/s

**Figure 4:**
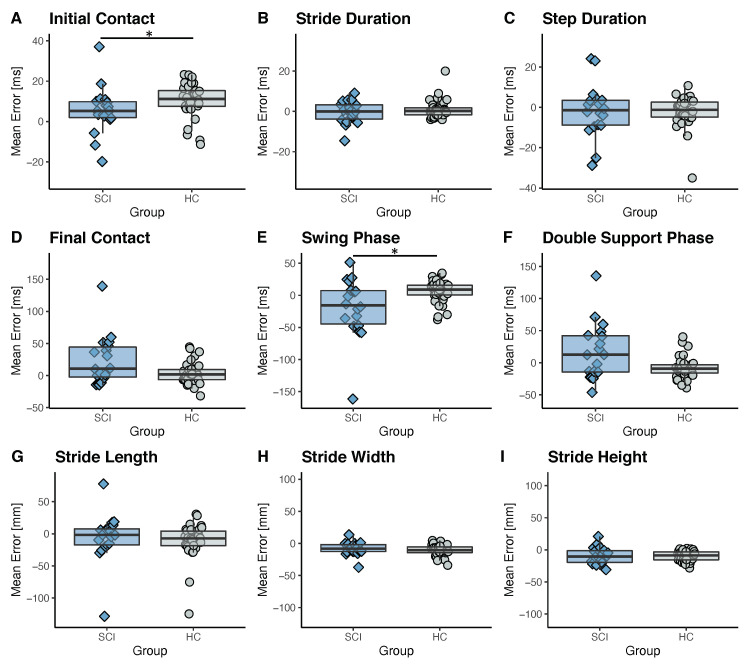
Mean error between the ground truth and sensor-derived gait parameters for the SCI patients and healthy controls (HC) walking on a treadmill at 0.5 m/s are shown. Boxes indicate 1st to 3rd quartile and the whiskers extend from the hinges to the largest/smallest value within 1.5 interquartile range. The black bar in the boxes displays the median. Significant differences between the two cohorts are indicated by * (*p* < 0.05).

The algorithm’s performance was analyzed using the same speed condition of 0.5 m/s for both the SCI patients and healthy controls. The mean error between the gait parameters derived from the gold standard and the inertial sensors attached to the ankles has been found to be similar for most of the parameters for the two cohorts, as shown in Figure 4. The values of the average and standard deviation for the different conditions can be found in Table A1 and Table A2 of the Appendix. A significant difference in the performance of the algorithm was obtained for the determination of the initial contact and swing phase. According to the results, the initial contact was detected more accurately for the SCI patients (5 ± 12 ms) than for the healthy controls (10 ± 9 ms). However, these results should be treated with caution because they are close to the resolution of the measurement method defined by the sampling rate of 200 Hz. The average and standard deviation of the error of the final foot contact detection (24 ± 39 ms) for the SCI participants are larger than in healthy controls, which resulted in larger errors and standard deviations for the estimation of the swing phase (−19 ± 48 ms) and the double support phase (17 ± 44 ms). However, only the swing phase was statistically significantly different between the two cohorts. Some of the SCI patients that participated in this study suffered from a so-called drop foot and thereby a pathological toe-off, which is typical, for example, for patients with a weak Tibialis Anterior [33] and explains the larger standard deviation for these parameters. For all the other gait parameters, no significant difference has been found between the two cohorts.

#### 3.3.2. Comparison of Errors between Slower and Preferred Walking Speed

**Figure 5:**
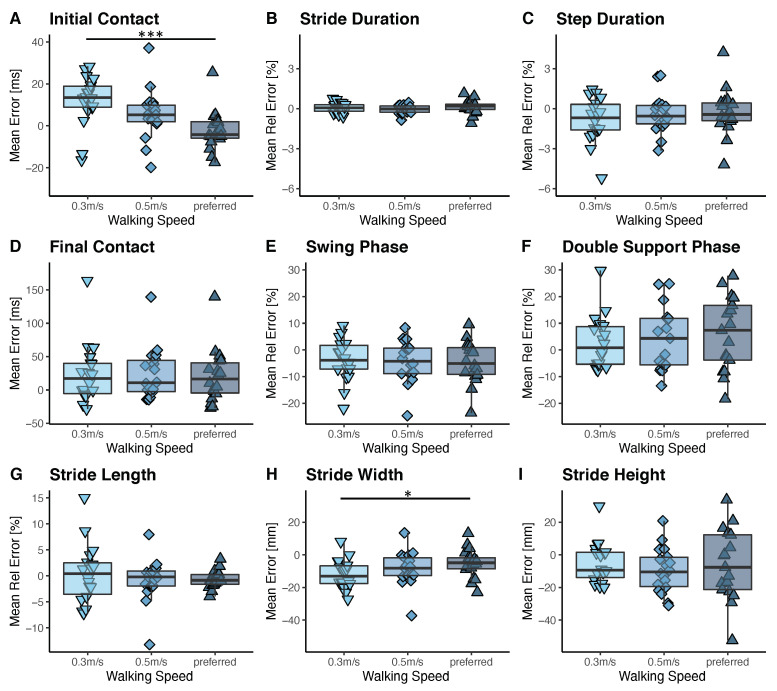
Mean error and mean relative error between ground truth and sensor-derived gait parameters for SCI patients walking on a treadmill at 0.3 m/s, 0.5 m/s, and their preferred speed. Boxes show 1st to 3rd quartile and the whiskers extend from the hinges to the largest/smallest value within 1.5 interquartile range. The black bar in the boxes displays the median. Significant differences between the different speed levels are indicated by * (*p* < 0.05) and *** (*p* < 0.001).

Speed had no effect on the algorithm performance in pathological gait for the majority of calculated parameters. The relative mean errors of the gait parameters are summarized in Figure 5 for the different walking speeds. Mean errors in [mm] are reported for parameters with small values, as a normalization would result in inflated values. This includes stride width and height. Moreover, mean errors in [ms] are reported for the initial and final foot contact detection, where normalization is not feasible. No statistically significant effect of speed was observed, except for the initial foot contact detection and the estimation of the stride width. Specifically, the initial contact was detected earlier than the ground truth for slower walking speeds. However, this had no significant effect on the estimation of any of the other temporal parameters. The stride width was more underestimated for slower walking speeds than for preferred walking speed. This can be explained by the drift in the double integration, which is larger for a longer stride duration due to the less frequent velocity updates and thus drift corrections. The values of the average and standard deviation for the different speed conditions can be found in Table A1 and Table A2 of the Appendix A.

#### 3.3.3. Overall Performance of the Algorithm

**Figure 6:**
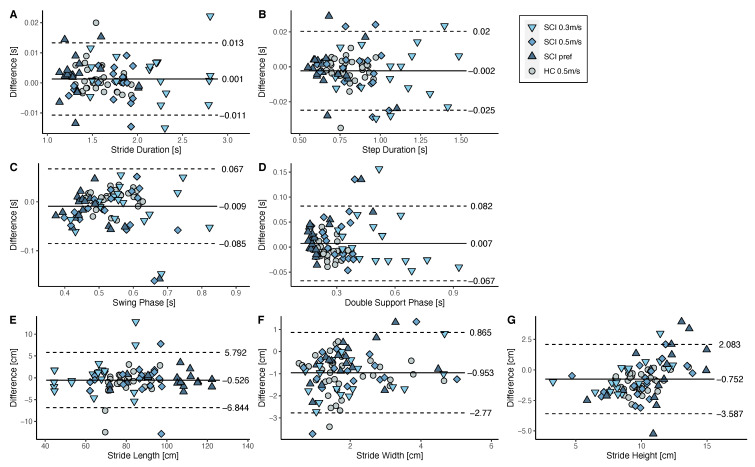
Bland-Altmann analysis of the differences between the sensor-derived gait parameters and the ground truth gait parameters for HC walking at 0.5m/s and SCI walking at 0.3m/s, 0.5m/s, and at their preferred speed. Differences are shown for the stride duration (**A**), step duration (**B**), swing phase (**C**), double support phase (**D**), stride length (**E**), stride width (**F**), and stride height (**G**). The black line indicates the mean difference between the two setups and the dashed lines represent the upper and lower limits of agreement.

The Bland–Altmann plots of the gait phases and spatial parameters of all speed conditions and cohorts are shown in Figure 6A–G. A maximum measurement bias of 9 ms was observed for the temporal parameters (stride duration, step duration, swing phase, and double support phase) and of −9.5 mm for the spatial parameters (stride length, width, and height). Therefore, there was a negligible small measurement bias for all the parameters. Moreover, there were negligible levels of dependency of the errors on parameter magnitude.

## 4. Discussion

This study proposes a method to extract spatio-temporal parameters from inertial sensors attached laterally above each ankle. In contrast to other algorithms, the proposed algorithm automatically uses personalized thresholds to detect each subject’s gait cycles and gait events.

The sensor-derived spatio-temporal parameters were validated for SCI patients walking at a steady state on an instrumented treadmill at 0.3 m/s, 0.5 m/s, and their preferred walking speed (0.76 ± 0.17m/s). The preferred walking speed of patients included in this study is similar to the average walking speed of 0.88 ± 0.06 m/s found by Barbeau et al. [34] for chronic SCI patients performing the six-minute walking test. Therefore, our study population is representative for this cohort. The algorithm’s performance for SCI patients was compared to that of healthy controls walking at 0.5 m/s to have an equivalent condition for the two groups. No extra or missing steps, and thus gait events, were detected in these two groups, which demonstrated the robustness of the proposed algorithm. Similarly, no missed or extra gait cycles were obtained for the different speed conditions in the SCI cohort. The results for the SCI patients walking at their preferred walking speed were compared to the performance of other algorithms from literature using shank-mounted inertial sensors for patients with a neurological disorder.

With the proposed algorithm, a mean error of −2 ± 9 ms and 20 ± 40 ms for detecting the initial (IC) and final (FC) foot contact have been obtained, respectively. Comparing these results to Jasiewicz et al. [20], our algorithm slightly outperforms their algorithm even for their cohort of SCI patients with regular footfall both for the IC (−15 ± 17 ms) and FC (28 ± 32 ms). However, the accuracy of the correct timing of the gait event detection is strongly dependent on how accurately the optical motion capture system and the sensor system are synchronized. Thus, comparing these results to literature is of less importance.

The mean errors of the stride (2 ± 7 ms) and step (−1 ± 13 ms) duration, which depend on the correct detection of the initial contact, were found to be similar to the results of Trojaniello et al. [15]. On average, their proposed algorithm resulted in a mean error of 0 ± 15 ms for the stride duration and 0 ± 18 ms for the step duration for the four cohorts investigated: elderly, hemiparetic, Parkinsonian, and choreic participants walking at different walking speeds. The lowest walking speed investigated was 0.61 ± 0.24 m/s with hemiparetic patients and the fastest walking speed was 1.49 ± 0.22 m/s with elderly participants. Thus, the range of speeds differs to the walking speeds evaluated in this study.

The results for the swing phase (−23 ± 44 ms) were slightly less accurate than the results of Salarian et al. for patients with Parkinson’s disease (5.9 ± 29.6 ms) [16]. This can be explained by the fact that SCI patients often suffer from abnormal footfalls, making the detection of the final foot contact difficult [20].

The stride length has been estimated with an error of −6 ± 17 mm, which corresponds to a mean relative error of −0.6%, vastly outperforming the results of Hundza et al. for Parkinson’s patients (110 ± 76.2 mm) [35]. The high achieved accuracy was surprising, especially considering the slow walking speeds investigated in this study. Slower walking speeds and the corresponding slower strides result in larger drifts, and thereby often larger errors. It seems that introducing an additional high-pass filter for abnormally long steps appropriately addresses this issue and significantly improves estimation accuracy.

To the best of our knowledge, the research articles available in the literature investigating the performance of sensor-based algorithms for slow walking speeds are very limited. This is especially important for the rehabilitation of SCI patients who are about to regain their walking function since they typically start to walk with speeds in the range of 0.34 m/s [23]. Forrest et al. demonstrated that a minimum of 0.44 m/s is necessary for limited community ambulation after an incomplete SCI [36]. Clinical application of sensor-based gait analysis aims to chart the recovery trajectory. Therefore, it is of paramount importance that algorithm performance is independent of walking speed and that the algorithm is validated for this range of low speeds. We here demonstrate that our algorithm meets these criteria for all typical spatio-temporal gait parameters, except initial foot contact detection and stride width estimation. However, the speed-dependent properties of these parameters did not carry over to other parameter estimations.

### Limitations and Future Work

The limitation of our validation method is that it was performed in a controlled setting, including steady-state straight walking on a treadmill only. There is an accumulation of evidence in healthy that treadmill walking can act as a rhythmic generator and reduce the variability of movement patterns [37]. Nevertheless, the basic kinematics are expected to remain similar [38]. As our algorithm was designed to perform well with high inter- and intra-subject variability, it seems plausible that it would function similarly well for steady-state over-ground walking. However, any application of the algorithm to walking including turns, uneven ground, or overcoming obstacles must be done with caution. In addition, our algorithm has been validated in nine SCI patients only due to the limited availability of SCI patients fulfilling the inclusion criteria. Nevertheless, these nine patients had varied sensory and motor impairments to cover a wide range of walking phenotypes, as demonstrated in the significant spread of ground-truth-derived gait parameters.

Future work of our group will focus on applying this algorithm to long-term data of SCI patients in daily life settings. Little is known on how walking during therapy time of SCI patients and the progress assessed by the clinical walking tests translate to daily life [39]. The benefit of monitoring daily life walking over clinical gait assessments was demonstrated already for healthy participants of different age groups [40], and patients with Parkinson’s disease [12]. Long-term measurements in a non-laboratory setting provide the possibility to assess the walking performance of SCI patients outside the therapy sessions. Furthermore, the walking performance can be compared to that during therapy time to objectively measure the progress and effect of therapeutic interventions on the daily life of the patients. This would be helpful not only as a research tool to measure the effect of novel therapeutic interventions and medications applied for SCI patients but could also be integrated into the daily clinical routine to track and motivate patients to translate their progress in therapy to leisure time. In addition, we believe that this algorithm could be applicable to other populations with atypical gait patterns. Therefore, we recently established a collaboration with a children’s hospital to investigate whether this algorithm can be generalized for use in children with neurological conditions. Similarly to SCI, this patient population suffers from diverse gait deficits depending on the neurological condition.

## 5. Conclusions

In this work, a novel algorithm that extracts typical spatio-temporal parameters from shank-mounted inertial sensors is proposed for the population of SCI patients. The spatio-temporal parameters were validated with 3D motion capture and force plates, a setup widely accepted as gold standard for gait analysis. For most of these parameters, the algorithm performed similarly well for both SCI patients and healthy controls, and the performance of the algorithm has been found to be robust for a wide range of walking speeds, including slow walking with a speed of 0.3 m/s. The results obtained are similar to those reported in literature for other patient populations with a neurological disorder. Due to the robustness over various walking speeds and the accuracy compared to gold standard measurements, we believe that the proposed algorithm is suitable for monitoring daily clinical routine and assessing the walking performance of SCI patients. This provides two clinically relevant perspectives: An extension of current clinical walking assessments to include markers of walking quality, and a high-density measurement of locomotor activity within and outside of clinical therapy. Both of these are necessary building blocks to achieve the leap to next-generation precision locomotor therapy.

## Figures and Tables

**Table 1:** Demographics (age, sex, height, and BMI) and clinical scores are listed of the spinal cord injury (SCI) patients who participated in the study. Within clinical scores, the completeness of injury (AIS), neurological level of injury (NLI), lower extremity motor score (LEMS), light touch, pin prick, and the mobility sub-score of the spinal cord independence measure (SCIM) are reported. The preferred walking speed as determined during the measurement is given for each patient.

ID	Age	Sex	Height	BMI	AIS	NLI	LEMS	Light Touch	Pin Prick	SCIM	Speed
	(yrs)	(m/f)	(cm)	(kg/m${}^{2}$)			L/R	L/R	L/R	Mobility	(m/s)
SCI01	69	f	167	16.1	D	C7	23/23	53/52	53/50	38	0.6
SCI02	48	m	184	24.8	D	L5	24/24	40/50	40/50	40	0.9
SCI03	58	f	163	24.5	D	T4	25/25	39/39	27/41	29	0.8
SCI04	58	f	170	19	D	T7	25/25	42/56	33/56	40	1
SCI05	55	m	169	19.3	D	C6	10/25	56/56	30/56	39	0.4
SCI06	70	m	170	23.2	D	T4-6	25/25	56/56	56/56	40	0.8
SCI07	66	m	170	25.6	D	T3	25/25	38/38	26/38	40	0.8
SCI08	54	m	184	31	D	T12	25/25	45/45	45/45	38	0.7
SCI09	58	m	178	20.8	D	C1	25/24	28/28	1/28	31	0.8

**Table 2:** Typical spatio-temporal parameters derived from the 3D motion capture and force plates are summarized for the SCI participants and healthy controls (HC). Values are given as mean ± standard deviation (intra-subject variability) for the different walking speeds.

Group	SCI			HC
Walking Speed [m/s]	0.3	0.5	0.76 ± 0.17	0.5
Number of included Gait Cycles	1493	1843	2254	2924
Number of excluded Gait Cycles	50	123	269	50
Stride Duration [s]	2.14 ± 0.47 (0.12)	1.69 ± 0.26 (0.08)	1.38 ± 0.26 (0.04)	1.60 ± 0.18 (0.05)
Step Duration [s]	1.07 ± 0.25 (0.08)	0.84 ± 0.14 (0.05)	0.69 ± 0.14 (0.03)	0.80 ± 0.09 (0.03)
Swing Phase [%]	28.7 ± 5.9 (3.1)	33 ± 4.2 (2.7)	35.5 ± 4.1 (2.0)	34.2 ± 1.9 (1.9)
Double Support Phase [%]	21.2 ± 5.1 (2.9)	16.9 ± 2.5 (1.9)	14.5 ± 2.8 (1.2)	15.8 ± 1.9 (1.4)
Stride Length [cm]	64.3 ± 14.3 (4.3)	84.3 ± 12.8 (4.4)	101.4 ± 16.2 (3.5)	80.2 ± 8.9 (3.1)
Stride Width [cm]	2.1 ± 1.3 (1.1)	2.1 ± 1.1 (1.2)	1.9 ± 1.0 (1.2)	1.9 ± 0.9 (0.6)
Stride Height [cm]	9 ± 2.2 (0.9)	10.1 ± 2.2 (0.6)	11.1 ± 2.5 (0.5)	10 ± 1.3 (0.5)

## Data Availability

The data are not publicly available due to ethical restrictions. Data sharing is not in accordance with the consent provided by the participants.

## Abbreviations

The following abbreviations are used in this manuscript:

SCI	Spinal Cord Injury
HC	Healthy Control
AIS	American Spinal Injury Association Impairment Scale
UEMS	Upper Extremity Motor Score
LEMS	Lower Extremity Motor Score
NLI	Neurological Level of Injury
SCIM	Spinal Cord Independence Measure
BMI	Body Mass Index
GRAIL	Gait Real-Time Analysis Interactive Lab
MSW	Mid-Swing
IC	Initial Contact
FC	Final Contact
MST	Mid-Stance

## Appendix A

**Table A1:** Mean error (me), its standard deviation (std), and the mean relative error (mre) between the temporal gait parameters derived from the GRAIL and the inertial sensors. The values are reported for SCI patients and healthy controls (HC) and the speed levels of 0.3 m/s, 0.5 m/s, and preferred (pref) walking speed.

Gait Parameter	Group	Speed	me [ms]	std [ms]	mre [%]
Initial Contact	SCI	0.3	12	12	
		0.5	5	12	
	HC	pref	−2	9	
		0.5	10	9	
Final Contact	SCI	0.3	24	45	
		0.5	24	39	
	HC	pref	20	40	
		0.5	4	17	
Stride Duration	SCI	0.3	2	8	0.1
		0.5	0	6	−0.1
	HC	pref	2	7	0.1
		0.5	1	5	0
Step Duration	SCI	0.3	−4	15	−0.8
		0.5	−2	13	−0.4
	HC	pref	−1	13	−0.3
		0.5	−2	8	−0.4
Swing Phase	SCI	0.3	−14	47	−3.8
		0.5	−19	48	−4.3
	HC	pref	−23	44	−4.7
		0.5	6	17	0.8
Double Support Phase	SCI	0.3	12	50	2.7
		0.5	17	44	5.8
	HC	pref	22	41	7.5
		0.5	−8	17	−3.5

**Table A2:** Mean error (me), its standard deviation (std), and the mean relative error (mre) between the spatial gait parameters derived from the GRAIL and the inertial sensors. The values are reported for SCI patients and healthy controls (HC) and the speed levels of 0.3 m/s, 0.5 m/s, and preferred (pref) walking speed.

Gait Parameter	Group	Speed	me [mm]	std [mm]	mre [%]
Stride Length	SCI	0.3	6	43	0.4
		0.5	−6	39	−0.7
	HC	pref	−6	17	−0.6
		0.5	−11	28	−1.5
Stride Height	SCI	0.3	−5	13	
		0.5	−10	14	
	HC	pref	−4	24	
		0.5	−10	8	
Stride Width	SCI	0.3	−12	9	
		0.5	−8	10	
	HC	pref	−5	9	
		0.5	−11	9	
